# Thermoregulatory and cardiovasculareffects of capsaicin application on human skin during dynamic exercise to temperate and warm conditions

**DOI:** 10.14814/phy2.14325

**Published:** 2019-12-27

**Authors:** Petros G. Botonis, Panagiotis G. Miliotis, Stylianos N. Kounalakis, Maria D. Koskolou, Nickos D. Geladas

**Affiliations:** ^1^ Section of Sport Medicine and Biology of Exercise School of Physical Education and Sport Science National and Kapodistrian University of Athens Athens Greece; ^2^ Faculty of Physical and Cultural Education Evelpidon Hellenic Army Academy Vari Greece

**Keywords:** core temperature, skin warm sensors, temperature and vascular regulation

## Abstract

Thermoregulatory and cardiovascular responses during cycling in temperate and warm environments without and with application of capsaicin on the skin were investigated. We hypothesized that regardless of environmental temperature, capsaicin application would activate heat loss mechanisms attenuating exercise‐induced rectal temperature (Tre) and blood pressure increase. Eight males cycled at 55% of their maximal aerobic power so long as to reach 38.2°C Tre at 20.8 ± 1.0°C and at 30.6 ± 1.1°C ambient temperatures twice: without (NCA) and with (CA) application of capsaicin patches (12 × 18 cm, 4.8 mg). Patches were applied on pectoralis major, trapezius and vastus lateralis muscles. Thermoregulatory (Tre, proximal‐distal skin temperature gradient, sweating rate), cardiovascular variables and oxygen uptake were continuously recorded. In both ambient conditions, during the first 14 min of exercise, the local vasoconstrictive tone as a function of the relative change in Tre was lower in CA than NCA (*p* < .05, *d* = 0.84–1.15). Further, sweating rate was higher and occurred at a lower Tre increase in CA compared to NCA (*p* = .03, *d = *0.6) resulting in extended time to reach 38.2°C Tre (*p* = .03, *d = *0.9). Moreover, oxygen consumption was higher in CA than in NCA (*p* < .001, *d = *0.8). Mean arterial pressure was lower during cycling in warm compared to temperate environment, but was unaffected by capsaicin. We conclude that activation of thermal sensors by capsaicin results in lower Tre rise during exercise, which is mediated through greater skin vasodilation along with higher rate and earlier onset of sweating. Nonetheless, capsaicin application has no extra effect on exercise cardiovascular responses.

## Graphical abstract

Capsaicin application on the human skin right before the initiation of cycling induced vasodilation and a higher rate and earlier onset of sweating resulting to a lower rectal temperature increase during exercise. These effects were unaffected by ambient temperature (temperate or warm). Capsaicin application had no extra effect on cardiovascular responses.

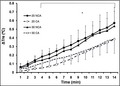

## 1 INTRODUCTION

It is well‐documented that heat‐sensitive and cold‐sensitive temperature receptor potential (TRP) channels significantly contribute to mammals’ temperature regulation (Caterina, 2007; Romanovsky et al., 2009). To date, a series of studies have proven that the application of chemical agonists of heat‐sensitive TRP vanilloid 1 (TRPV1; e.g., capsaicin) or cold‐sensitive TRP Melastatin8 (TRPM8) channels (e.g., menthol) on the skin can be used as a novel approach for investigating the integrative mechanisms affecting humans’ temperature (Botonis, Geladas, Kounalakis, Cheouveim, & Koskolou, 2017; Botonis, Kounalakis, Cherouveim, Koskolou, & Geladas, 2018; Kounalakis, Botonis, Koskolou, & Geladas, 2010) and vascular regulation (Botonis, Miliotis, Koskolou, & Geladas, 2019; Ives et al., 2017).

A recent study in humans (Botonis et al., 2019), confirming previous observations in animals (Donnerer & Lembeck, 1983; Jansco‐Cabor, Szolcsanyi, & Jancso, 1970), demonstrated a significant rectal temperature decline during resting conditionas a result of capsaicin application on subjects’ skin. Along these lines, studies conducted in vivo have shown that TRPV1 channels contribute to thermal hyperaemia (Wong & Fieger, 2010) accompanied by cutaneous active vasodilation (Wong & Fieger, 2012). Yet, to date, it remains unknown whether the proven vasodilatory and heat‐dissipating effects of capsaicin can be extrapolated during exercise, where cutaneous vasoconstrictive tone and sweating are activated (Johnson, 2010).

Alongside its thermoregulatory properties, skin TRPV1 activation through capsaicin application may be promising for promoting blood pressure homeostasis (McCarty, DiNicolantonio, & O'Keefe, 2015). It was recently demonstrated that along with heat loss activation, capsaicin induces arterial hypotension in resting subjects exposed to 30°C (Botonis et al., 2019). Moreover, the hypotensive effect of capsaicin has been verified during static exercise protocols in animals (Ichiyama, Ragan, Bell, & Iwamoto, 2002) and humans (Dawson, Walser, Jafarzadeh, & Stebbins, 2004; Vianna, Fernandes, Barbosa, Teixerira, & Nobrega, 2018). Notwithstanding the increasing accumulation of research underlining the cardioprotective effects of TRPV1 activation (Mc Carty et al., 2015) and its influence on blood pressure at rest and during static exercise, the cardiovascular effects of TRPV1 stimulation through capsaicin application during dynamic whole‐body exercise have never been determined.

Therefore, this study aimed at exploring thermoregulatory and cardiovascular responses during cycling in a temperate (20°C) and in a warm (30°C) environment without and with application of capsaicin on the skin (5% of the skin surface area) of human subjects. We hypothesized that, regardless of environmental temperature, capsaicin application on the skin would activate heat loss mechanisms, which, in turn, would attenuate exercise‐induced rectal temperature and blood pressure rise; this response would be probably due to thermal sensors excitation induced by capsaicin.

## 2 METHODS

### 2.1 ETHICAL approval

This study was approved by the Human Research Ethics Committee of National and Kapodistrian University of Athens, Greece (1053/16/5/2018) and was performed in accordance with the latest instructions of the *Declaration of Helsinki*. Participants provided oral and written informed consent before enrolment in the study.

### 2.2 Participants

Eight healthy male participants (age: 20.4 ± 2.1 years, body mass: 80.5 ± 6.1 kg, stature: 180.4 ± 6.6 cm, body fat: 10.7 ± 3.7%, maximal oxygen consumption: 42.4 ± 4.2 ml kg min^−1^, aerobic power: 286 ± 37.4 watt) volunteered to participate in this study. All participants were nonsmokers, physically active (2–5 days/week), not acclimatized to hot environment or diagnosed with any cardiovascular, respiratory or metabolic disease at the time of the study.

### 2.3 Preliminary testing

Prior to the main trials, the participants visited the laboratory where they were initially familiarized with the experimental protocol and equipment and were tested for a possible undesirable capsaicin reaction by applying a patch containing the substance on the skin area of the forearm. On a subsequent visit, participants reported to the laboratory and body fat was assessed by taking measurements of several skinfolds (chest, thigh, triceps, subscapular, suprailiac, abdominal and axillary) and using the equation of Jackson and Pollock (1978) for the calculation of body fat percentage. Subsequently, each participant performed an incremental protocol on a cycle ergometer (Lode) with increments of 30 W per minute to exhaustion. Resting values were measured for a 5‐min period preceding the exercise bout. The initial workload was 50 W and thetarget pedaling rate 70–80 rpm.

### 2.4 Main trial

For 2 days prior to their first experimental trial, participants were asked to record their diet and physical activity and were instructed to replicate these habits before each experiment, to ensure similar levels of body energy sources and hydration. In addition, participants had to abstain from strenuous exercise, caffeine and alcohol consumption the day before the experiments. Participants attended the laboratory at the same time of the day on four occasions separated by 2–7 days. On the experimental day, after participants voiding their bladder and having their body mass measured (Bilance Salus), a rectal thermistor was inserted 13–15 cm beyond the anal sphincter. Subsequently, while being instrumented, they sat and relaxed for 20 min. The participants were then instructed to cycle so long as to reach a rectal temperature of 38.2°C in temperate (20°C) or warm (30°C) environmental temperature and without (NCA) or with (CA) capsaicin application on the skin. In all experimental conditions, exercise intensity corresponded to 55% of participants’ maximal aerobic power. In CA condition, four commercially available patches (12 × 18 cm), each one impregnated with of 4.8 mg capsaicin (C_18_H_27_NO_3_), were applied on four different parts of the body known to exhibit different thermal sensitivity (Cotter & Taylor, 2005), so as to obtain sampling from a range of thermal sensitity points. In particular, one patch was applied on pectoralis major muscle, one was applied on trapezius muscle and the other two patches were applied on the two vastus lateralis muscles. Thus, by selecting these areas we tried to be unbiased in terms of skin thermosensitivity. All application sites had been previously shaved. In NCA, before initiation of the experimental trial, there was a 2‐min initial pre‐experimental period of collecting reference data at the respective environmental temperature (20°C or 30°C). In CA condition, there was no time interval between this pre‐experimental resting phase and consecutive application of capsaicin patches on the body. Two‐min resting values with capsaicin patches on the skin were then recorded. Exercise data collection started immediately after the two min of rest. The environmental temperature, controlled in an environmental chamber, was either warm (30.6 ± 1.1°C, 40.8 ± 1.1% relative humidity) or temperate (20.8 ± 1.0°C, 40.6 ± 0.9% relative humidity). The testing order was counterbalanced in terms of capsaicin application and environmental temperature. All experiments were conducted during the spring (between March and June) or autumn season (between October and November) and at the same time of the day.

### 2.5 Measurements

#### 2.5.1 Thermoregulatory measurements

Throughout the experiment, skin temperatures (forearm, fingertip, major pectoralis and trapezius muscle) were recorded additionally to rectal temperature. Chest (major pectoralis) and back (trapezius muscle) skin thermistors were applied on skin areas free of capsaicin and the mean skin temperature was calculated as the average of forearm, chest and back temperatures. All temperatures were recorded continuously with thermistors (SS7, BIOPAC Systems Inc.) connected to a mobile unit (TEL 100D, BIOPAC Systems Inc.); they were fast response thermistors having, according the manufacturer, ±0.2°C interchangeability over the range of 31°C–45°C with response time 0.6 s. These responses were confirmed in the lab in 3 different water temperatures ranging from 30°C to 45°C before and half the way through the experiments**.** For each measurement, the signal was transferred from the mobile unit (TEL 100D, BIOPAC Systems Inc.) to a signal processing unit (MP 100A, BIOPAC System, Inc.) and then stored on a computer. The difference in temperature between forearm and fingertip (Tskf‐f) was calculated as an index of skin vasomotor tone with higher difference suggesting lower local blood flow (Keramidas, Geladas, Mekjavic, & Kounalakis, 2013; Rubinstein & Sessler, 1990).

Minute mass flow of local secreted sweat rate of the forearm was measured continuously during the experiment at a frequency of 200 Hz by means of a ventilated capsule, positioned on a 5.72‐cm^2^ area of skin of the right forearm and ventilated at a rate of 2 L/min with air at room temperature. The temperature and humidity of air entering and exiting the capsule were measured with thermocouples (TSD 202A, BIOPAC, Systems Inc.) and capacitance hygrometers (Delta), respectively. Sweating rate (SwR) was estimated from the difference between the temperature and the humidity of inflowing and outflowing air. Temperature and humidity sensors were appropriately calibrated before their use.

#### 2.5.2 Cardiorespiratory measurements

Cardiovascular variables (blood pressure, total peripheral resistance, cardiac output, stroke volume and heart rate) were continuously recorded noninvasively via a photoplethysmometer with the cuff attached on the middle finger of the right hand (Finometer 2003, FMS). Thus stroke volume is computed from the arterial pressure wave, with continuous nonlinear corrections for variations in aortic diameter, compliance and impedance during the arterial pulsation (Wesseling, Jansen, Settels, & Schreuder, 1993). Integrating the aortic flow waveform per beatprovides left‐ventricular SV and cardiac output (CO) is calculated by multiplying SV with heart rate. The Modelflow technique provides stroke volume values using a nonlinear three element ((a) impedance of the aorta (b) total arterial compliance (c) peripheral vascular resistance) model of the aortic input impedance and computes an aortic flow waveform from a peripheral arterial pressure signal. The first two elements depend on the elastic properties of the aorta varying in a nonlinear manner with distending pressure and compute the aortic flow waveform, while the third element is calculated for each beat by the model stimulation and updated. The modelflow method is reliable and provides CO values precisely from arterial pressure compared to thermodilution technique with small error, where the mean deviation from thermodilution CO was 2% and *SD* 8% (Jellema, Imholz, Oosting, Wesseling, & Van Lieshoout, 1999; Wesseling et al., 1993).

Before each experiment, Finometer device was automatically calibrated for pressure, distance of finger sensor from the heart level and detection of sound derived from “return to arm flow”, according to the manufacturer's standards. Moreover, oxygen uptake ($\dot{V}$O_2_) was measured continuously with a metabolic cart (MedGraphics, CPX‐D), which was calibrated with two different gas mixtures (atmospheric air and prime gas of 15.02% O_2_ and 5.03% CO_2_ with uncertainty 1%) and a 3‐L syringe before each testing; the metabolic cart used has high validity (experimental error: −2.6 ± 4.5% and 1.7 ± 2.7%, for VO_2_ and VCO_2_, respectively) (Miodownik, Carlon, Ferri, & Melendez, 2000) and has been shown to provide reliable results (Prieur et al., 1998). In each experimental condition, cardiac work was calculated by multiplying the values of mean arterial pressure by heart rate (Gobel, Norstrom, Nelson, Jorgensen, & Wang, 1978).

#### 2.5.3 Rating of thermal sensation and perceived exertion

Ratings of thermal whole‐body sensation were reported by the subjects using a visual analogue scale questionnaire (1, cold; 3, cool; 5, slightly cool; 7, neutral, 9; slightly warm, 11; warm, 13; hot) (ASHRAE, 1966, modified) every 5 min. At the same intervals, perceived exertion was reported by the subjects using the Borg category scale (Borg, 1982). Participants were familiarized with the scales prior to testing.

### 2.6 Statistical analysis

Before using parametric tests for the analysis of the data, the assumption of normality was verified using the Shapiro–Wilk test. All variables were found with normal distribution and, consequently, a three‐way analysis of variance (ANOVA) for repeated measures on three factors (environmental temperature × capsaicin application × time) was used to define the overall differences in each variable. A Tukey test was employed to allocate post hoc specific differences. The rate of rectal temperature rise was the averaged slope, which derived from the linear regression analysis of rectal temperature course for each subject. In addition, deviation from linearity using the least squares linear regression method was used to determine the rectal threshold for the initiation of sweating. As a measure of effect size, the Cohen's *d* was calculated dividing the difference between sample means by the standard deviation of difference scores. Values of 0.20, 0.50 and above 0.80 were considered as small, medium and large, respectively. Except for thermal sensation and perceived exertion, all variables were analyzed up to the 14th minute of exercise in order to have equal number of subjects for statistical analysis (*n* = 8). Data are presented as mean ± standard deviation (*SD*), unless indicated otherwise. Significance level was set at .05.

## 3 RESULTS

### 3.1 Rectal temperature and exercise time

**Figure 1:**
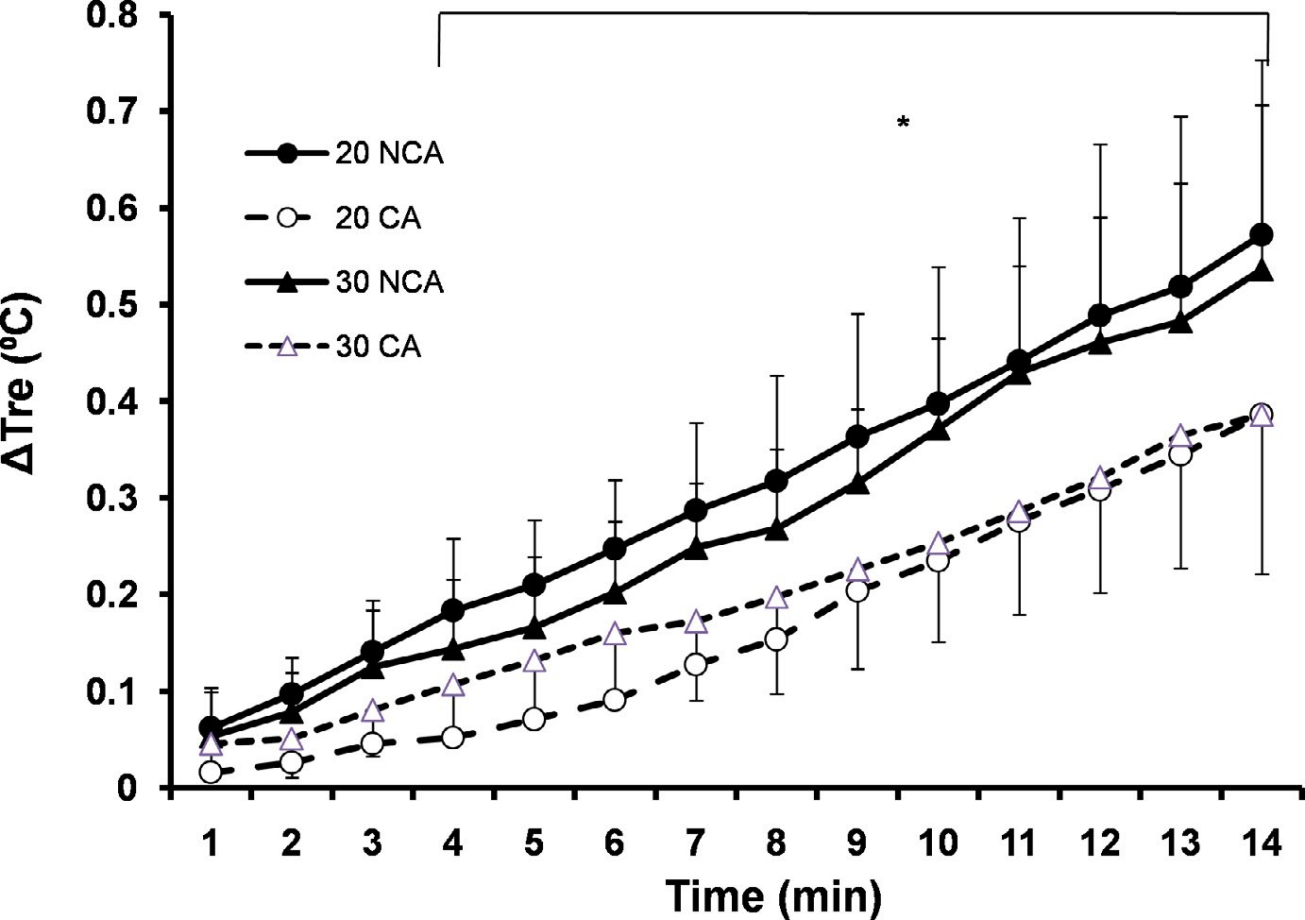
Relative changes (mean ± *SD*) in rectal temperature (ΔTre) during 14 min of cycling attemperate (20°C) and warm (30°C) environment with (CA) and without (NCA) capsaicin application (*N* = 8). *Significant difference between CA and NCA (*p* < .001)

Initial pre‐exercise values for rectal temperature were similar among conditions (Table 1). The rate of rise to reach 38.2°C in rectal temperature was not affected by ambient temperature, but it was slower (0.03 ± 0.01°C/min) in capsaicin than in noncapsaicin condition (0.04 ± 0.02°C/min) (*F*(1,7) = 4.71, *p* < .05, *d* = 0.5). Accordingly, the cycling time to reach 38.2°C was longer by 5.4 ± 6.1 min in CA compared to NCA (*F*(1,7) = 7.95, *p* = .03, *d* = 0.9, Table 2). Furthermore, from the 4th up to the 14th min of exercise, rectal temperature was unaffected by ambient temperature, but it was lower in capsaicin than in noncapsaicin condition (*F*(1,7) = 49.34, *p* < .001, *d* = 1.7) (Figure 1).

**Table 1:** Pre‐exercising, idle values of selected thermoregulatory and cardiovascular variables during exposure to 20°C and 30°C without (ΝCA) and with (CA) capsaicin application on the skin (*N* = 8)

	20°C		30°C		Effect of temperature	Effect of capsaicin
	NCA	CA	NCA	CA	*p*	*p*
Tre (°C)	37.2 (0.1)	37.2 (0.1)	37.2 (0.2)	37.2 (0.2)	.96	.70
Mean Tsk (°C)	33.2 (2.5)	33.2 (0.8)	34.4 (1.2)	34.7 (0.9)	.02	.85
ΔTsk f‐f (°C)	2.4 (3.0)	0.6 (2.2)	2.2 (4.0)	0.8 (3.7)	.99	.05
SwR (g cm^2^ min^−1^)	−0.5 (2.1)	−0.0.9 (3.1)	−1.6 (3.1)	−0.9 (3.1)	.71	.70
MAP (mm Hg)	103.2 (6.0)	99.4 (7.0)	95.8 (4.4)	94.4 (8.0)	.02	.28
SYS (mm Hg)	142.5 (9.1)	134.1 (7.4)	136.9 (14.9)	131.7 (9.5)	.28	.14
DIA (mm Hg)	81.0 (4.5)	79.1 (6.0)	74.5 (2.7)	73.5 (7.3)	.01	.45
HR (beats/min)	72.9 (8.0)	73.3 (6.9)	74.6 (6.8)	74.0 (8.2)	.61	.93
SV (l/min)	110.8 (11.4)	104.7 (9.2)	109.9 (14.3)	104.5 (17.8)	.83	.19
CO (l/min)	8.0 (1.0)	7.6 (0.6)	8.4 (1.6)	7.6 (1.3)	.57	.02
TPR (MU)	0.79 (1.0)	0.81 (0.1)	0.70 (0.1)	0.86 (0.4)	.72	.19
TS (a.u)	4.9 (1.8)	5.3 (1.4)	8.3 (1.0)	7.9 (1.0)	.00	1.00

Note

All values are mean (*SD*).

Abbreviations: CO, cardiac output; DIA, diastolic pressure; HR, heart rate; MAP, mean arterial pressure; SV, stroke volume; SYS, systolic pressure; TPR, total peripheral resistance; Tre, rectal temperature; TS, thermal sensationΔTsk f‐f, difference in temperature between forearm and fingertip; Μean Tsk, mean skin temperature.

**Table 2:** Selected thermoregulatory and cardiovascular responses up to the 14th min of exercise and cycling time to reach 38.2°C in rectal temperature during exercise to 20°C and 30°C without (ΝCA) and with (CA) capsaicin application on the skin (*N* = 8). All values are mean (*SD*)

	20°C		30°C		Effect of temperature	Effect of capsaicin
	NCA	CA	NCA	CA	*p*	*p*
Mean Tsk (°C)	34.0 (1.9)	33.6 (0.9)	35.7 (1.0)	35.4 (0.5)	.00	.30
MAP (mm Hg)	123.2 (7.0)	125.9 (9.6)	111.8 (9.6)	113.0 (12.7)	.00	.57
SYS (mm Hg)	181.2 (10.1)	178.3 (17.7)	164.2 (18.4)	165.6 (17.2)	.02	.84
DIA (mm Hg)	92.2. (6.0)	95.5 (6.9)	82.5 (8.3)	83.9 (11.6)	.00	.23
HR (beats/min)	138.2 (9.6)	141.8 (6.9)	142.4 (6.0)	141.7 (6.1)	.23	.05
SV (l/min)	138.3 (17.5)	130.6 (18.1)	143.8 (24.3)	138.1 (23.8)	.21	.05
CO (l/min)	19.1 (2.5)	18.6 (3.3)	20.5 (3.6)	19.5 (3.4)	.11	.12
TPR (MU)	0.5 (0.2)	0.4 (0.1)	0.4 (0.1)	0.4 (0.1)	.02	.92
Cycling time (min)	28.3 (7.7)	31.1 (7.9)	23.5 (6.1)	31.4 (7.1)	.35	.03

Abbreviations: CO, cardiac output; DIA, diastolic pressure; HR, heart rate; MAP, mean arterial pressure; SV, stroke volume; SYS, systolic pressure; TPR, total peripheral resistance; Μean Tsk, mean skin temperature.

### 3.2 Skin temperatures and sweating rate

Initial pre‐exercise values for the mean skin temperature were higher in warm compared to temperate environment, but similar between capsaicin and noncapsaicin condition (Table 1). The mean skin temperature increased over time and it was higher in 30°C than in 20°C (*F*(1,7) = 27.74, *p* = .001, *d = *1.47), but no differences were detected between CA and NCA conditions (Table 2).

**Figure 2:**
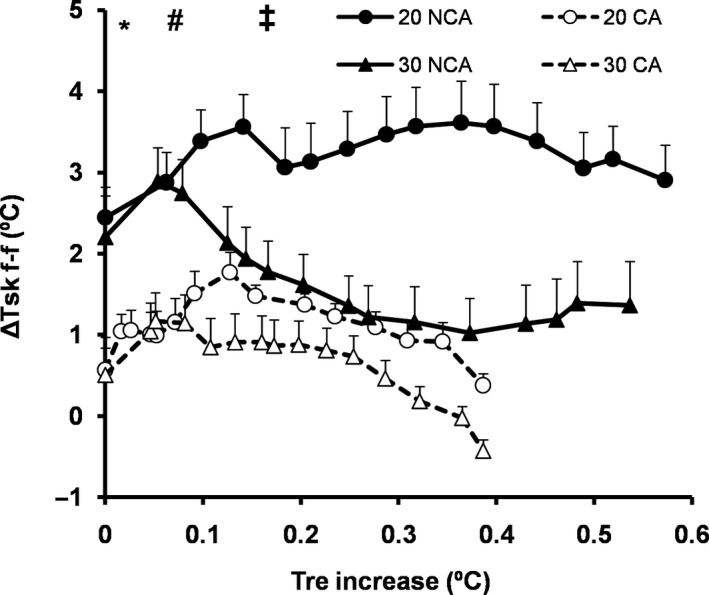
Changes (mean ± *SD*) in vasomotor tone (temperature difference between forearm and fingertip (ΔΤskf‐f)) as a function of the relative change in rectal temperature (Tre) during 14‐min cycling at temperate (20°C) and warm (30°C) environment with (CA) and without (NCA) capsaicin application on the skin (*N* = 8). *Significant difference between CA and NCA at 0°C (*p* < .01, *d* = 1.15). ^#^Significant difference between CA and NCAat 0.1°C of Tre increase (*p* = .02, *d* = 0.93). ^‡^Significant difference between CA and NCA at 0.2°C of Tre increase (*p* = .03, *d* = 0.84)

**Figure 3:**
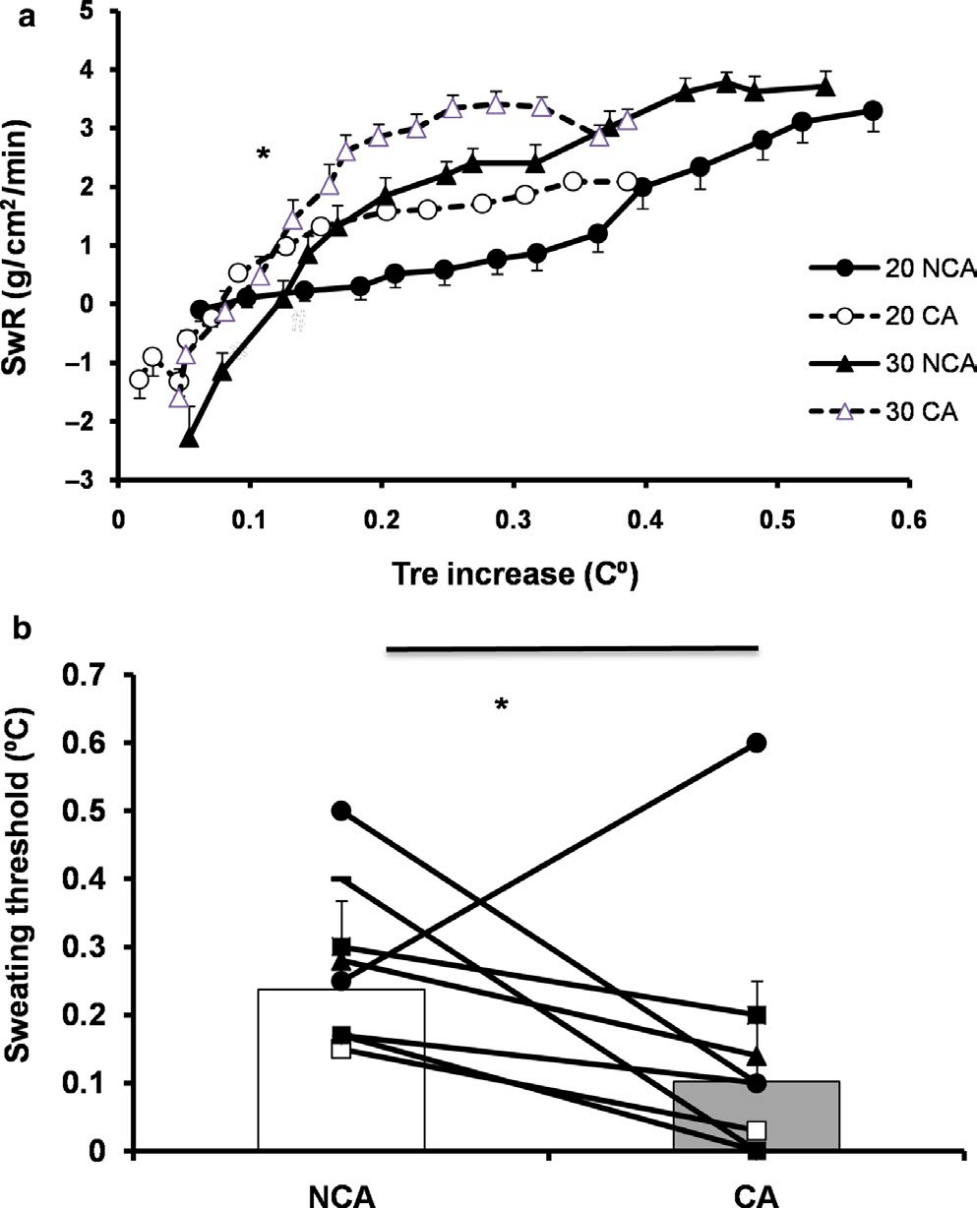
Sweating rate (SwR; mean ± *SE*) as a function of the relative change in rectal temperature (Tre) during 14‐min cycling at temperate (20°C) and warm (30°C) environment with (CA) and without (NCA) capsaicin application on the skin (*N* = 8) (Figure 3a) and individual and mean (*SD*) values of rectal temperature at which the threshold of sweating occurred (Figure 3b) during 14‐min cycling at temperate (20°C) and warm (30°C) environmentwith (CA) and without (NCA) capsaicin application on the skin (*N* = 8). (a) *Significant difference between CA and NCA (*p* = .05, *d = *0.5). (b) *Significant difference between CA and NCA (*p* = .03, *d = *0.6)

Initial pre‐exercise values (Table 1) for the index of local vasomotor tone (ΔTskf‐f) were similar between temperate and warm conditions, but they were lower in capsaicin than in noncapsaicin condition (*p* = .05, *d* = 0.64). The index of local vasomotor tone (ΔTskf‐f) as a function of the relative change in rectal temperature increase showed similar values between 30°C and 20°C, but lower values (*p* < .05, *d* = 0.84–1.15) in CA than in NCA (Figure 2). Additionally, initial values for sweating rate were similar among experimental conditions (Table 1). Moreover, the analysis of sweating rate as a function of rectal temperature increase showed that participants in CA displayed significantly higher sweating compared to NCA condition (main effect: *F*(1,7) = 5.90, *p* = .05, *d = *0.48, Figure 3a). Accordingly, the threshold of sweating occurred at lower rectal temperature in CA (0.1 ± 0.1°C) than in NCA condition (0.2 ± 0.1°C) (*F*(1,7) = 6.84, *p* = .03, *d = *0.6) (Figure 3b).

### 3.3 Oxygen uptake

**Figure 4:**
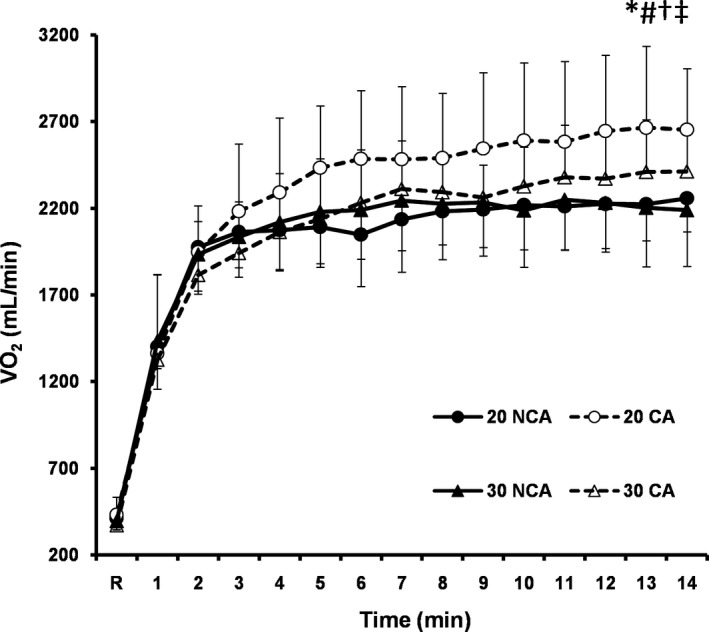
Mean (*SD*) values of oxygen uptake (VO_2_) during14min of cycling at temperate (20°C) and warm (30°C) environment with (CA) and without (NCA) capsaicin application on the skin (*N* = 8). *Significant difference between CA and NCA (*p* < .01, *d = *0.8). ^#^Significant difference between 20°C CA and 20°C NCA (*p* = .01, *d = *1.4). ^†^Significant difference between 20°CA and 30°C NCA (*p* = .02, *d = *1.3). ^‡^Significant difference between 20°C CA and 30°C CA (*p* = .04, *d = *1.0)

Oxygen uptake was increased over time and it was significantly higher in capsaicin than in noncapsaicin condition (*F*(1,7) = 24.70, *p* = .01, *d = *0.8) (Figure 4). In addition, a significant interaction (ambient temperature X capsaicin) was found (*p* = .03). The post hoc analysis revealed that oxygen uptake was higher in 20°C CA compared to 20°C NCA (*p* = .01, *d = *1.4), 30°C NCA (*p* = .02, *d = *1.3) and 30°C CA (*p* = .04, *d = *1.0), whereas no differences were detected among the other trials (Figure 4).

### 3.4 Arterial pressure

Initial pre‐exercising values for mean arterial pressure were lower in warm than in temperate environment, but similar between capsaicin and noncapsaicin condition (Table 1). Mean arterial pressure showed a progressive decline over time (*F*(14,98) = 38.52, *p* < .001) and was lower when participants exercised in 30°C than in 20°C (*F*(1,7) = 16.98, *p* < .01, *d = *1.0), but remained unaffected by capsaicin application (Table 2). Accordingly, the initial pre‐exercising values for systolic pressure were no different among experimental conditions, except for a tendency for lower systolic pressure (*p* = .14) found with capsaicin as compared to noncapsaicin (Table 1). The respective values for diastolic pressure, however, were lower in warm than in temperate environment and no differences were shown between capsaicin and noncapsaicin condition (Table 1). Both systolic and diastolic pressure were reduced over time (*F*(14,98) = 19.41, *p* < .001 and *F*(14,98) = 31.14, *p* < .001, respectively) and were lower in 30°C than in 20°C (*F*(1,7) = 8.51, *p* = .02 and *F*(1,7) = 20.89, *p* < .01, *d = *0.8 and *d = *1.1, respectively). Nonetheless, none of them were influenced by capsaicin application (Table 2).

### 3.5 Total peripheral resistance (TPR) and cardiac output

Initial, pre‐exercise values for cardiac output were similar between environmental temperatures, but they were lower in capsaicin than in noncapsaicin condition (Table 1). During exercise, total peripheral resistance was lower in warm compared to temperate ambient temperature (*F*(1,7) = 8.41, *p* = .02, *d* = 0.65), but similar between capsaicin versus*.* noncapsaicin conditions (Table 2). Νo differences were observed in stroke volume and heart rate between 30°C and 20°C, but cardiac output tended to be higher (*p* = .11) at 30°C (Table 2). Stroke volume was lower and heart rate was higher in capsaicin compared to noncapsaicin condition (Table 2) resulting in slightly (*p* = .12) lower cardiac output in capsaicin condition. Moreover, the cardiac work was higher in temperate (17,434.8 ± 1688.8 mmHg beats^−1^ min^−1^) compared to warm condition (1593.11 ± 1,528 mmHg beats^−1^ min^−1^) (*F*(1,7) = 9.13, *p* = .02, *d* = 0.81) and in capsaicin (16,929 ± 2,038 mmHg beats^−1^ min^−1^) than in noncapsaicin condition (16,438 ± 1,452 mmHg beats^−1^ min^−1^) (*F*(1,7) = 7.87, *p* = .03, *d* = 0.36).

### 3.6 Ratings of thermal sensation and perceived exertion

**Figure 5:**
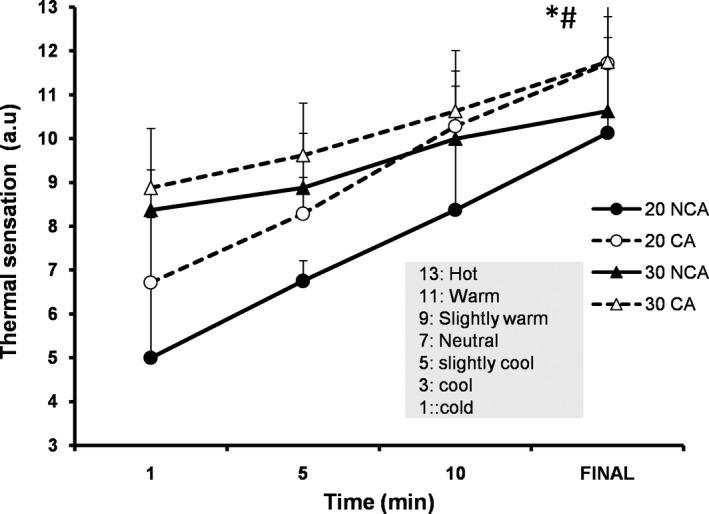
Mean (*SD*) thermal sensation values recorded at 1st, 5th, 10th and at the final min of exercise at temperate (20°C) and warm (30°C) environment with (CA) and without (NCA) capsaicin application on the skin (*N* = 8). *Significant difference between 20°C and 30°C (*p* < .01, *d = *1.7). ^#^Significant difference between CA and NCA (*p* = .02, *d = *1.0)

Initial values for the rate of thermal sensation were higher in 30°C compared to 20°C, but similar between capsaicin versus. noncapsaicin condition (Table 1). The rate of thermal sensation increased over time (*F*(4,28) = 90.96, *p* < .001) and the mean value was higher during exercise at 30°C (9.8 ± 1.6 a.u) compared to 20°C (8.2 ± 2.4 a.u) (*F*(1,7) = 33.03, *p* < .001, *d = *1.7). Similarly, the average value of thermal sensation was higher in capsaicin (9.5 ± 2.1 a.u) than in noncapsaicin condition (8.5 ± 2.2 a.u) (*F*(1,7) = 8.76, *p* = .02, *d = *1.0) (Figure 5). Moreover, the rate of perceived exertion increased over time (*F*(3,21) = 26.74, *p* < .001), but remained unaffected by ambient temperature as well as by capsaicin application.

## 4 DISCUSSION

We investigated the effects of capsaicin application on the skin (5% of the skin surface area) during dynamic whole‐body exercise on thermoregulatory and cardiovascular responses at temperate and warm conditions. The principal findings of the present study were: (a) regardless of ambient temperature, capsaicin application before the initiation of exercise reduced heat gain during exercise due toa greater heat loss indicated by a lower estimated vasoconstrictive tone of the skin as well as to an earlier onset and higher rate of sweating; in turn, both these responses ended in a slower rise of rectal temperature during exercise, and (b) the higher heat dissipation observed after application of capsaicin was not accompanied by lessened blood pressure increase during exercise, as it was anticipated based on previous data at rest.

In accordance with the literature underlying the influence of TRP temperature‐sensitive channels on human's temperature regulation (Gillis, House, & Tipton, 2010; Lee, Nakao, Bakri, & Tochichara, 2012), the present study indicates that TRPV1 excitation through capsaicin affects human's temperature homeostasis, even during exercise. Consistent with our initial hypothesis, we observed that regardless of environmental temperatures, capsaicin application reduced the exercise‐induced heat gains. As a consequence, the cycling time needed for subjects’ rectal temperature to reach 38.2°C was increased by 5 min approximately in capsaicin compared to the noncapsaicin condition. The present findings are in accordance with our previous work in resting humans (Botonis et al., 2019), indicating that capsaicin application on the skin (5% of the skin surface area) induces significant rectal temperature decline as a result of TRPV1 activation. Extending this knowledge, the present study showed that during cycling, the estimated vasoconstrictive tone was lower and the sweating rate was higher, with its initiation occurring at lower rectal temperature rise, after capsaicin application, thus resulting in a slower rectal temperature increase. The increased sweating observed in capsaicin condition is likely due to capsaicin‐induced activation of nitric oxide (NO) (Randhawa & Jaggi, 2017), which results in augmented vasodilation and thermal hyperaemia (Wong & Fieger, 2010). In addition, NO synthase has been suggested to be integral in modulating both sweating and cutaneous dilation in humans (Welch, Nakao, Bakri, & Tochichara, 2009). Although skin blood flow was not measured in the present study, the considerable reduction of the estimated local vasoconstrictive tone after capsaicin treatment implies higher skin blood flow, as the two variables are related during exercise (Keramidas et al., 2013), and therefore reduced exercise‐induced heat gains. Interestingly, despite the observed similarity in skin temperatures between conditions (capsaicin vs. noncapsaicin), it appears that capsaicin acts as a chemical at the small peripheral patches applied on the skin of the subjects (5% of the skin surface area) stimulating warm (43°C) TRPV1 receptors and thus inducing central whole body thermoregulatory and cardiovascular responses. Additionally, it has been postulated that TRPV1 activation modulates vascular function and induces sympatholysis by opposing a‐adrenergic receptor‐mediated vasoconstriction and potentiating vasorelaxation (Ives et al., 2017). In this context, the dilatory effect of capsaicin has been well documented (Randhawa & Jaggi, 2017) and might be linked with calcitonin gene‐related peptide release (Brain, Williams, Tippins, Morris, & Maclntyre, 1985), which stimulates NO release in the endothelial cells (Randhawa & Jaggi, 2017). To date, a number of studies have demonstrated that NO synthase is an important modulator of sweat rate (Fujii et al., 2014, 2016; Louie, Fujii, Meade, & Kenny, 2016; Welch et al., 2009). Interestingly, Welch et al. (2009) showed that the administration of NOS inhibitors attenuated sweating in participants who exercised in the heat.

Unexpectedly to findings at rest (Botonis et al., 2019), heat production (oxygen uptake) was higher during exercise with than without capsaicin application. However, this finding is in accordance with experiments in resting rats (Hursel & Westerterp‐Plantega, 2010), which have shown that capsaicin may increase heat production by enhancing catecholamine secretion from the adrenal medulla, mainly through activation of central nervous system. Experiments in exercising animals have shown that the effects of capsaicin on metabolism are probably related to the down‐regulation of the uncoupling protein 3 (UCP3), which was accompanied with an increase in the mitochondrial ATP production (Faraut et al., 2007). Despite the increased heat production observed in capsaicin condition, the overall heat loss was higher in the former condition, thus causing a slower rise of rectal temperature.

We also observed that heat dissipation (estimated cutaneous vasodilation and sweating) occurring as a consequence of capsaicin application was not accompanied by lower increase of blood pressure during exercise. This finding is in contrast to previous studies conducted both in animals (Ichiyama et al., 2002; Nelson, Ragan, Ichiyama, & Iwamoto, 2004) and humans (Dawson et al., 2004), who received epidermal capsaicin administration. Nonetheless, our results are in accordance with the respective ones of Vianna et al. (2018), who observed similar increase of blood pressure during handgrip exercise after the application of capsaicin on the skin compared to control condition. Several reasons may explain this interesting finding. First of all, different methodological approaches concerning the dosage of capsaicin have been applied. We presently used commercially available patches (12 × 18 cm; 5% of the skin surface area) popular for their analgesic properties containing 4.8 mg of capsaicin, whereas previous studies in humans (Dawson et al., 2004) showing hypotensive effects had used greater dosage of capsaicin. Additionally, it is known that the hypotensive effect of capsaicin is related to the capsaicin‐induced desensitization of group III and IV muscle afferent fibers (Dawson et al., 2004; Nelson et al., 2004). However, this was not the case in the present study, as the course of arterial blood pressure was similar between capsaicin and noncapsaicin conditions. Therefore, it could be alleged that a possible activation of group ΙΙΙ and IV muscle afferent fibers as a result of a metabolically demanding exercise counteracts the possible role of capsaicin on the same fibers. In fact, the present exercise protocol was different from previous ones (Dawson et al., 2004; Vianna et al., 2018), wherein static exercise was applied.

In the present study, also, two different environmental temperatures were used to investigate the effects of capsaicin on thermoregulatory and cardiovascular responses during exercise. In this direction, a previous study (Botonis et al., 2019) had shown interactive effects of capsaicin and environmental temperature. Nonetheless, the present results indicate that ambient temperature does not influence the magnitude of capsaicin effects.

Due to the burning effect of capsaicin, it was not possible to keep the experimental design blind. Thus, the placebo effect cannot be excluded. Moreover, we acknowledge that our control experiments were conducted without placebo patches. This was due to the fact that making a control patch similar in view with the real capsaicin patch without the burning effect was difficult. It is well known that regional variations in temperature sensitivity exist across the human body (Nadel, Mitchell, & Stolwijk, 1973). We acknowledge that, in the present study, capsaicin patches were applied on selected anatomical sites (quadriceps, major pectoralis, trapezius muscle), which represent different thermal sensitivity (Filingeri, 2016), with the lower limbs exhibiting lower thermal sensitivity than the central/upper regions of the body (Cotter & Taylor, 2005; Nadel et al., 1973; Stevens, Marks, & Simonson, 1974). It is still unknown whether application of capsaicin on different body regions has similar thermoregulatory effects.

The current study demonstrates that regardless of environmental condition (temperate or warm) thermal sensors activation by capsaicin application on the human skin (5% of the skin surface area) reduces heat gains during dynamic exercise and results in a slower increase of rectal temperature. These effects are mediated through lower estimated skin vasoconstriction, and higher, but also earlier onset of sweating. Nonetheless, in the present setting, capsaicin did not influence the course of arterial pressure, thus indicating that during submaximal cycling corresponding to 55% of maximal aerobic power, the activation of group III and IV muscle afferent fibers counteracts the opposite effect of capsaicin and as such the effect of capsaicin on arterial pressure is insignificant. More research is needed to explore the effects of capsaicin during dynamic exercise in a different exercise intensity spectrum as well as during the post exercise state.

## CONFLICT OF INTEREST

None of the authors has any conflict of interest.

## AUTHOR CONTRIBUTIONS

*Conception or design of the work*: P.G.B., N.D.G. *Acquisition, data analysis, or interpretation of the data work*: P.G.B., P.G.M., S.N.K. M.D.K. N.D.G. *Drafting of the work or revising it critically for important intellectual content*: P.G.B., P.G.M., S.N.K. M.D.K. N.D.G. All authors approved the final version of the manuscript. All authors listed qualify for authorship, and have approved the final manuscript. All authors agree to be accountable for all aspects of the work in ensuring that questions related to the accuracy or integrity of any part of the work are appropriately investigated and resolved. All authors acknowledge their role in the study and agree to be held accountable for their participation.
